# Costs of Treating Onasemnogene Abeparvovec‐Xioi‐Induced Liver Injury

**DOI:** 10.1002/prp2.70134

**Published:** 2025-06-12

**Authors:** Andrej Belančić, Branislava Raičević, Ivana Stević, Dinko Vitezić, Slobodan M. Janković

**Affiliations:** ^1^ Department of Basic and Clinical Pharmacology and Toxicology Faculty of Medicine, University of Rijeka Rijeka Croatia; ^2^ Faculty of Medical Sciences, University of Kragujevac Kragujevac Serbia; ^3^ Department of Social Pharmacy and Pharmaceutical Legislation Faculty of Pharmacy, University of Belgrade Belgrade Serbia

**Keywords:** drug safety, gene therapy, liver injury, onasemnogene abeparvovec‐xioi, pharmacoeconomics, spinal muscular atrophy

## Abstract

Aims were to reveal types of onasemnogene abeparvovec‐xioi (OA)‐induced liver injury, their treatment patterns, utilization of healthcare, and treatment costs. This study employed secondary research to analyze OA‐induced liver injury using data from the EudraVigilance database, published case reports, cohort studies, and clinical trials. The extracted data were analyzed to define real‐life clinical entities that could be clearly outlined as syndromes resulting from the OA‐induced liver injury, and further used in guiding the development of healthcare utilization matrices. Serbian healthcare costs were calculated by multiplying utilization figures by local unit prices, converted to Euros using exchange rates and adjusted by price level indices. A spreadsheet model with uniform distributions simulated costs for 1000 virtual patients, providing mean values and standard deviations for Serbia and the EU. From 1566 adverse event reports in the EudraVigilance database following OA therapy, 231 were hepatobiliary disorders, predominantly hypertransaminasaemia (30.7%; 71/231). Liver injury largely manifested as mild‐to‐moderate biochemical abnormalities, rarely progressing to severe complications, and was effectively managed with corticosteroid therapy. Economic analysis highlights the manageable burden of OA‐induced liver injury. In the EU, mild‐to‐moderate cases cost €823.7, while severe cases average €1638.6. Medication costs range from €26.8 for prednisone to €695.4 for severe cases requiring additional immunosuppressive agents like tacrolimus and mycophenolate mofetil. To conclude, OA‐induced liver injury, though notable, is clinically manageable with immunosuppressive therapy and rarely causes severe complications like encephalopathy or liver failure. Its modest costs do not undermine OA's cost‐effectiveness, supporting its transformative role in spinal muscular atrophy treatment.

## Graphical abstract

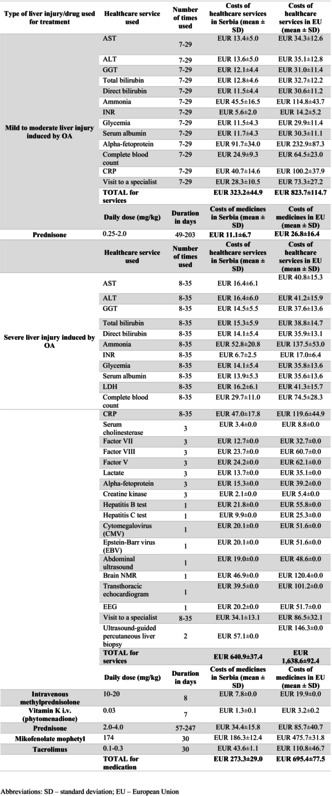

## 1 Introduction

Gene therapy of symptomatic infantile‐onset spinal muscular atrophy (SMA) with onasemnogene abeparvovec‐xioi (OA) has recently achieved spectacular results, which led to its marketing authorization and widespread use [1]. The transgene missing in patients with SMA is carried by a parvovirus called associated viral vector (AAV) which after intravenous administration enters various cells, including hepatocytes [2]. However, one adverse event associated with OA emerged as surprisingly frequent, requiring routine prophylaxis with corticosteroids: hepatotoxicity. There are several ways how OA may damage hepatocytes, causing an increase in serum levels of bilirubin, aminotransferases, and other liver enzymes: overexpression of the transgene, cell‐mediated immune response, and direct toxic effect of OA [3]. Depending on the severity of hepatocyte injury, a patient may have an adverse event of mild, moderate, or severe extent, ranging from just an increase in serum levels of hepatic enzymes without symptoms to severe hepatitis and liver failure [3].

Appearance and treatment of OA‐induced liver injury may place additional workload and financial burden on healthcare systems which already pay high costs of the treatment itself [4]. However, the extent of healthcare utilization and costs generated by treatment of OA‐induced liver injury are currently unknown, and sometimes a matter of guessing [5]. The aim of this study was to reveal types of OA‐induced liver injury, their treatment patterns, utilization of healthcare, and treatment costs.

## 2 Methods

The study was designed as a secondary research based on the extraction of data about OA gene therapy‐induced liver injuries from the EudraVigilance (EV) database (containing information on suspected adverse reactions to medicines that have been authorized or are being studied in clinical trials in the European Economic Area [EEA]) [6] and published case reports, case series, cohort studies, and clinical trials.

The EudraVigilance Data Analysis System (EVDAS) is a web‐based application developed by the European Medicines Agency (EMA) to support pharmacovigilance activities in the EU/EEA, with a primary focus on signal detection and the evaluation of individual case safety reports (ICSRs). EVDAS is part of the broader EV system, which is the EMA's comprehensive platform for managing and analyzing reports of suspected adverse drug reactions (ADRs) to medicines that are either authorized or under investigation in the EEA. These reports are submitted by national competent authorities (NCAs) and marketing authorisation holders (MAHs), and they include both serious and non‐serious ADRs occurring within the EEA. The EV database facilitates pharmacovigilance through the structured use of the Medical Dictionary for Regulatory Activities (MedDRA) and incorporates analytical tools such as EVDAS. One key feature of EVDAS is the inclusion of disproportionality measures, which support signal detection by identifying drug‐event pairs reported more frequently than expected. It is important to note that the data in EudraVigilance consist of suspected ADRs, meaning no formal clinical assessments are conducted to confirm a causal relationship between the drug and the reported event [7, 8, 9].

In the first step of the present study, the data were extracted separately for the active substance (OA) and trade name (“Zolgensma”) and were analyzed to define real‐life clinical entities that could be clearly outlined as syndromes resulting from the OA‐induced liver injury. The next step consisted of the construction of healthcare utilization matrices for each of the defined entities, based on the extracted data. In the third step, the utilization figures were multiplied by the unit prices of the healthcare services [10] and medicines [11] recognized by the Republic Health Insurance Fund of Serbia; the prices were converted from Republic of Serbia Dinars (RSD) to Euro using the mean exchange rate of the National Bank of Serbia on July the 1st, 2024 [12]. In the final step, the costs of treating OA‐induced liver injury in Serbia were used as the basis for the calculation of average costs of services and medicines in European Union (EU) countries: Serbian costs were divided by the Serbian price level index for healthcare spending (output‐based), and then multiplied by the average price level index of EU countries [13].

Since the utilization figures were presented as medians and ranges of values in the publications, in Microsoft Excel, a spreadsheet model for the calculation of costs was constructed using input with uniform distributions of the utilization data instead of single values. The author S.J. created a macro in Visual Basic to simulate a model on a virtual cohort. The model was simulated using a virtual cohort of 1000 patients to obtain the mean values and standard deviations of the healthcare costs for both Serbia and the EU. The results are presented in tables.

## 3 Results

In the EudraVigilance database [6] from 2019 to 2024, there were 813 and 753 spontaneous reports of adverse events related to the treatment of symptomatic infantile‐onset SMA with OA gene therapy and Zolgensma, respectively, where 127 and 104 of them were classified as hepatobiliary disorders, respectively. The number of reported individual cases for main reaction groups, along with their share in total reported cases, is presented in Table 1. The structure of hepatobiliary adverse events related to the OA is shown in Table 2. Apart from the fact that the causality of reported adverse events was not proven, limited data in the records precluded the understanding of the real clinical picture, its severity, treatment, and outcomes.

**TABLE 1:** Spontaneous reports per reaction groups reported for onasemnogene abeparvovec and Zolgensma gene therapy of infantile‐onset spinal muscular atrophy.

Reaction groups	Onasemnogene abeparvovec	Zolgensma
	Frequency (%^a^)	Frequency (%^a^)
Blood and lymphatic system disorders	192 (23.62)	176 (23.37)
Cardiac disorders	55 (6.77)	53 (7.04)
Congenital, familial, and genetic disorders	17 (2.09)	16 (2.12)
Ear and labyrinth disorders	5 (0.62)	4 (0.53)
Endocrine disorders	3 (0.37)	3 (0.40)
Eye disorders	9 (1.11)	9 (1.20)
Gastrointestinal disorders	222 (27.31)	204 (27.09)
General disorders and administration site conditions	275 (33.83)	255 (33.86)
Hepatobiliary disorders	127 (15.62)	104 (13.81)
Immune system disorders	16 (1.97)	13 (1.73)
Infections and infestations	144 (17.71)	140 (18.59)
Injury, poisoning, and procedural complications	29 (3.57)	29 (3.85)
Investigations	473 (58.18)	437 (58.03)
Metabolism and nutrition disorders	91 (11.19)	87 (11.55)
Musculoskeletal and connective tissue disorders	30 (3.69)	29 (3.85)
Neoplasms benign, malignant, and unspecified (incl cysts and polyps)	2 (0.25)	2 (0.27)
Nervous system disorders	72 (8.86)	67 (8.90)
Pregnancy, puerperium, and perinatal conditions	—	—
Product issues	—	—
Psychiatric disorders	49 (6.03)	48 (6.37)
Renal and urinary disorders	28 (3.44)	25 (3.32)
Reproductive system and breast disorders	1 (0.12)	1 (0.13)
Respiratory, thoracic, and mediastinal disorders	127 (15.62)	121 (16.07)
Skin and subcutaneous tissue disorders	39 (4.80)	38 (5.05)
Social circumstances	—	—
Surgical and medical procedures	—	—
Vascular disorders	38 (4.67)	37 (4.91)

*Note:* Reports are derived from the EudraVigilance database (cut‐off date: 13th November 2024).

^a^ Calculated on the total number of all reported cases of 813 and 753 for Onasemnogene Abeparvovec and Zolgesma, respectively.

**TABLE 2:** Spontaneous reports of hepatobiliary adverse events related to onasemnogene abeparvovec and Zolgensma gene therapy of infantile‐onset spinal muscular atrophy.

Adverse event	Onasemnogene abeparvovec	Zolgensma
	Frequency (%)	Frequency (%)
Acute hepatic failure	8 (6.30)	5 (4.81)
Autoimmune hepatitis	1 (0.79)	1 (0.96)
Cholangitis	1 (0.79)	—
Cholestasis	3 (2.36)	3 (2.88)
Chronic hepatitis	1 (0.79)	1 (0.96)
Drug‐induced liver injury	5 (3.94)	4 (3.85)
Gallbladder enlargement	3 (2.36)	3 (2.88)
Hepatic cytolysis	9 (7.09)	9 (8.65)
Hepatic failure	4 (3.15)	3 (2.88)
Hepatic fibrosis	1 (0.79)	—
Hepatic function abnormal	9 (7.09)	6 (5.77)
Hepatic steatosis	1 (0.79)	1 (0.96)
Hepatitis	8 (6.30)	7 (6.73)
Hepatomegaly	3 (2.36)	3 (2.88)
Hepatosplenomegaly	1 (0.79)	—
Hepatotoxicity	5 (3.94)	3 (2.88)
Hyperbilirubinaemia	2 (1.57)	2 (1.92)
Hypertransaminasaemia	39 (30.71)	32 (30.77)
Immune‐mediated hepatic disorder	1 (0.79)	1 (0.96)
Jaundice	5 (3.94)	4 (3.85)
Liver disorder	9 (7.09)	9 (8.65)
Liver injury	3 (2.36)	3 (2.88)
Liver tenderness	1 (0.79)	1 (0.96)
Ocular icterus	2 (1.57)	2 (1.92)
Subacute hepatic failure	2 (1.57)	1 (0.96)
Total	127 (100)	104 (100)

*Note:* Reports are derived from the EudraVigilance database (cut‐off date: 13th November 2024).

After analysis of published case reports (*n* = 2) [14, 15], case series (*n* = 2) [16, 17], cohort studies (*n* = 3) [18, 19, 20] and clinical trials (*n* = 2) [21, 22] (Table S1) involving treatment of the SMA with OA, two clinical entities of hepatobiliary adverse events could have been singled out: mild to moderate liver injury and severe liver injury. The data on utilization of healthcare resources for the treatment of OA‐induced mild to moderate and severe liver injury were extracted from the publications, and costs calculated by multiplying utilization figures with prices of healthcare services [10] and medicines [11] recognized by the Republic Health Insurance Fund of Serbia. The utilization and cost data are shown in Table 3.

**TABLE 3:** Utilization and the costs of healthcare resources for treatment of onasemnogene abeparvovec‐xioi (OA)–induced mild to moderate and severe liver injury.

**Type of liver injury/drug used for treatment**	**Healthcare service used**	**Number of times used**	**Costs of healthcare services in Serbia (mean ± SD)**	**Costs of healthcare services in EU (mean ± SD)**
Mild to moderate liver injury induced by OA	AST	7–29	EUR 13.4 ± 5.0	EUR 34.3 ± 12.6
	ALT	7–29	EUR 13.6 ± 5.0	EUR 35.1 ± 12.8
	GGT	7–29	EUR 12.1 ± 4.4	EUR 31.0 ± 11.4
	Total bilirubin	7–29	EUR 12.8 ± 4.6	EUR 32.7 ± 12.2
	Direct bilirubin	7–29	EUR 11.5 ± 4.4	EUR 30.6 ± 11.2
	Ammonia	7–29	EUR 45.5 ± 16.5	EUR 114.8 ± 43.7
	INR	7–29	EUR 5.6 ± 2.0	EUR 14.2 ± 5.2
	Glycemia	7–29	EUR 11.5 ± 4.3	EUR 29.9 ± 11.4
	Serum albumin	7–29	EUR 11.7 ± 4.3	EUR 30.3 ± 11.1
	Alpha‐fetoprotein	7–29	EUR 91.7 ± 34.0	EUR 232.9 ± 87.3
	Complete blood count	7–29	EUR 24.9 ± 9.3	EUR 64.5 ± 23.0
	CRP	7–29	EUR 40.7 ± 14.6	EUR 100.2 ± 37.9
	Visit to a specialist	7–29	EUR 28.3 ± 10.5	EUR 73.3 ± 27.2
	Total for services		EUR 323.2 ± 44.9	EUR 823.7 ± 114.7
	**Daily dose (mg/kg)**	**Duration in days**	**Costs of medicines in Serbia (mean ± SD)**	**Costs of medicines in EU (mean ± SD)**
Prednisone	0.25–2.0	49–203	EUR 11.1 ± 6.7	EUR 26.8 ± 16.4
	**Healthcare service used**	**Number of times used**	**Costs of healthcare services in Serbia (mean ± SD)**	**Costs of healthcare services in EU (mean ± SD)**
Severe liver injury induced by OA	AST	8–35	EUR 16.4 ± 6.1	EUR 40.8 ± 15.3
	ALT	8–35	EUR 16.4 ± 6.0	EUR 41.2 ± 15.9
	GGT	8–35	EUR 14.5 ± 5.5	EUR 37.6 ± 13.6
	Total bilirubin	8–35	EUR 15.3 ± 5.9	EUR 38.8 ± 14.7
	Direct bilirubin	8–35	EUR 14.1 ± 5.4	EUR 35.9 ± 13.1
	Ammonia	8–35	EUR 52.8 ± 20.8	EUR 137.5 ± 53.0
	INR	8–35	EUR 6.7 ± 2.5	EUR 17.0 ± 6.4
	Glycemia	8–35	EUR 14.1 ± 5.4	EUR 35.8 ± 13.6
	Serum albumin	8–35	EUR 13.9 ± 5.3	EUR 35.6 ± 13.6
	LDH	8–35	EUR 16.2 ± 6.1	EUR 41.3 ± 15.7
	Complete blood count	8–35	EUR 29.7 ± 11.0	EUR 74.5 ± 28.3
	CRP	8–35	EUR 47.0 ± 17.8	EUR 119.6 ± 44.9
	Serum cholinesterase	3	EUR 3.4 ± 0.0	EUR 8.8 ± 0.0
	Factor VII	3	EUR 12.7 ± 0.0	EUR 32.7 ± 0.0
	Factor VIII	3	EUR 23.7 ± 0.0	EUR 60.7 ± 0.0
	Factor V	3	EUR 24.2 ± 0.0	EUR 62.1 ± 0.0
	Lactate	3	EUR 13.7 ± 0.0	EUR 35.1 ± 0.0
	Alpha‐fetoprotein	3	EUR 15.3 ± 0.0	EUR 39.2 ± 0.0
	Creatine kinase	3	EUR 2.1 ± 0.0	EUR 5.4 ± 0.0
	Hepatitis B test	1	EUR 21.8 ± 0.0	EUR 55.8 ± 0.0
	Hepatitis C test	1	EUR 9.9 ± 0.0	EUR 25.3 ± 0.0
	Cytomegalovirus (CMV)	1	EUR 20.1 ± 0.0	EUR 51.6 ± 0.0
	Epstein–Barr virus (EBV)	1	EUR 20.1 ± 0.0	EUR 51.6 ± 0.0
	Abdominal ultrasound	1	EUR 19.0 ± 0.0	EUR 48.6 ± 0.0
	Brain NMR	1	EUR 46.9 ± 0.0	EUR 120.4 ± 0.0
	Transthoracic echocardiogram	1	EUR 39.5 ± 0.0	EUR 101.2 ± 0.0
	EEG	1	EUR 20.2 ± 0.0	EUR 51.7 ± 0.0
	Visit to a specialist	8–35	EUR 34.1 ± 13.1	EUR 86.5 ± 32.1
	Ultrasound‐guided percutaneous liver biopsy	2	EUR 57.1 ± 0.0	EUR 146.3 ± 0.0
	Total for services		EUR 640.9 ± 37.4	EUR 1638.6 ± 92.4
	**Daily dose (mg/kg)**	**Duration in days**	**Costs of medicines in Serbia (mean ± SD)**	**Costs of medicines in EU (mean ± SD)**
Intravenous methylprednisolone	10–20	8	EUR 7.8 ± 0.0	EUR 19.9 ± 0.0
Vitamin K i.v. (phytomenadione)	0.03	7	EUR 1.3 ± 0.1	EUR 3.2 ± 0.2
Prednisone	2.0–4.0	57–247	EUR 34.4 ± 15.8	EUR 85.7 ± 40.7
Mikofenolate mophetyl	174	30	EUR 186.3 ± 12.4	EUR 475.7 ± 31.8
Tacrolimus	0.1–0.3	30	EUR 43.6 ± 1.1	EUR 110.8 ± 46.7
	Total for medication		EUR 273.3 ± 29.0	EUR 695.4 ± 77.5

Abbreviations: EU, European Union; SD, standard deviation.

Based on Serbian costs of healthcare services and medications, average costs in European Union (EU) countries were calculated by applying the price level index for healthcare spending (output‐based): Serbian index was 0.39 in 2014 when compared to the 1.0 average index of EU countries [13]. Average costs of treating OA‐induced liver injury in EU countries are shown in the last column of Table 3.

## 4 Discussion

OA represents a ground‐breaking therapeutic advancement for SMA type 1, offering transformative outcomes with a single‐dose administration [1, 18, 19, 20, 22]. However, hepatotoxicity remains a frequently reported adverse event, raising questions about its clinical burden and economic implications [3, 4]. In this study, we provide real‐world pharmacovigilance evidence (RWE) on the types of OA‐induced liver injury, its management principles, and associated costs. The present analyses are particularly valuable in rare diseases, offering insights into disease‐modifying therapy individualization and indirect treatment comparisons in terms of clinical outcomes and economic aspects. This enhances clinical decision‐making, particularly in cases where primary treatments prove inadequate or lead to adverse drug reactions.

To deduce, the findings confirm that OA‐induced liver injury, while relatively frequent, remains clinically manageable and does not significantly jeopardize the overall cost‐effectiveness of OA therapy. From our analysis of spontaneous reports in the EudraVigilance database, 127 and 104 of 813 and 753 spontaneous reports of adverse events following OA and Zolgensma therapy, respectively, were hepatobiliary disorders, with hypertransaminasaemia (~30%) being the most frequent [6]. Importantly, our results confirm that liver injury primarily manifests as biochemical abnormalities (mild‐to‐moderate), with minimal progression to severe complications such as encephalopathy or acute liver failure requiring hospitalization. Even in severe cases, liver dysfunction was effectively treated with immunosuppressive therapies—namely, oral prednisone and intravenous methylprednisolone, with occasional use of additional immunomodulators (e.g., tacrolimus, mycophenolate mofetil). This highlights the efficacy of corticosteroid therapy in resolving OA‐related hepatotoxicity without significant healthcare resource utilization [14, 15, 16, 17, 18, 19, 20, 21, 22].

The economic analysis further supports the manageable burden of OA‐induced liver injury. For mild‐to‐moderate cases, treatment costs in the EU average €823.7, primarily driven by laboratory monitoring and specialist visits, while severe cases incur €1638.6. Moreover, the costs of medicines in the EU for the treatment of mild‐to‐moderate liver injury induced by OA average €26.8 for prednisone, while for severe liver injury, the costs include €695.4 in total, driven by the use of prednisone, intravenous methylprednisolone, and additional immunosuppressive agents such as tacrolimus and mycophenolate mofetil, as detailed in Table 3.

Notably, hospitalization was largely avoided, underscoring the cost‐efficiency of outpatient management [6, 14, 15, 16, 17, 18, 19, 20, 21, 22]. Overall, findings confirm that liver toxicity, while common, adds only a modest financial burden relative to the overall cost of OA therapy and is unlikely to significantly impact its favorable cost‐effectiveness profile.

Belančić et al. [23] previously highlighted the necessity of pharmacoeconomic analyses—including cost‐effectiveness and cost‐utility evaluations—particularly for orphan medicines with exceptionally high costs. Willingness‐to‐pay thresholds specific to orphan drugs are essential to ensure equitable access for SMA patients. Our findings contribute to this discussion by confirming that the costs of managing OA‐induced liver injury are modest relative to its clinical benefit, further supporting its value proposition. Importantly, such evaluations must incorporate the impact of new knowledge and updated RWE on therapeutic strategies, ensuring effective resource allocation towards treatments offering the highest benefit [23].

### 4.1 Strengths and Limitations

The present study provides a robust analysis of OA‐induced liver injury, synthesizing real‐world adverse event reports with published evidence to quantify both clinical and economic burdens. By applying healthcare cost indices, we extrapolate findings across diverse healthcare systems, offering insights relevant to clinicians, policymakers, and payers. However, the reliance on spontaneous reporting systems introduces limitations, including underreporting and incomplete clinical details, the absence of the denominator data for the population exposed, as well as the inclusion of non‐healthcare professional reports. Moreover, potential duplicate reporting under both the substance and brand name, although likely infrequent in real‐world practice, may lead to some overestimation of adverse event frequency and associated costs. Additionally, while our analysis confirms short‐ to medium‐term outcomes, long‐term implications of OA‐induced liver injury remain uncertain. Variability in treatment approaches across real‐world settings may further impact cost estimates.

## 5 Conclusions

OA‐induced liver injury, though a relatively notable adverse event, remains clinically manageable with immunosuppressive therapy, rarely leading to severe complications such as encephalopathy or liver failure requiring hospitalization. The associated costs, even for severe liver injury, are modest in the context of OA's overall economic impact. Thus, liver toxicity does not significantly compromise the regular cost‐effectiveness of OA, reinforcing its role as a transformative therapy for SMA.

## Author Contributions

Project administration, A.B., D.V., S.M.J.; methodology, S.M.J., A.B.; investigation, S.M.J., A.B., B.R., I.S.; writing – original draft, A.B., S.M.J.; writing – review and editing, A.B., B.R., I.S., D.V., S.M.J.; conceptualization, S.M.J., A.B.; supervision, D.V., S.M.J. All authors have read and agreed to the published version of the manuscript.

## Ethics Statement

The authors have nothing to report.

## Conflicts of Interest

The authors declare no conflicts of interest.

## Supporting information


**Table S1.** Summary of hepatotoxicity cases reported in studies on onasemnogene abeparvovec‐xioi treatment for spinal muscular atrophy.

## Data Availability

Data sharing is possible upon reasonable request sent to the corresponding author.
