# Supplementary material for: Costs of Treating Onasemnogene Abeparvovec‐Xioi‐Induced Liver Injury
Source: Pharmacol Res Perspect. 2025 Jun 12;13(3):e70134. doi: 10.1002/prp2.70134 (PMC12163186; doi:10.1002/prp2.70134)
Supplement: Supplementary file 1 — Table S1. Summary of hepatotoxicity cases reported in studies on onasemnogene abeparvovec‐xioi treatment for spinal muscular atrophy. [file PRP2-13-e70134-s001.docx]

**Supplementary table 1.** Summary of hepatotoxicity cases reported in studies on onasemnogene abeparvovec-xioi treatment for spinal muscular atrophy

| **Reference** | **Number of Cases** | **Number of Cases with Any Hepatotoxicity AEs** | **% of Cases with Any Hepatotoxicity AEs** |
| --- | --- | --- | --- |
| Nigro et al. (2023) [14] | 1 | 1 | 100.00% |
| Funck et al. (2023) [15] | 1 | 1 | 100.00% |
| Pongsakornkullachart et al. (2024) [16] | 8 | 8 | 100.00% |
| Friese et al. (2021) [17] | 8 | 8 | 100.00% |
| D’Silva et al. (2022) [18] | 21 | 12 | 57.14% |
| Nevmerzhitskaya et al. (2021) [19] | 10 | 10 | 100.00% |
| Gowda et al. (2024) [20] | 99 | 87 | 87.88% |
| Chand et al. (2021) [21] | 325 | 69 | 21.23% |

**Note:** Data from Reference 22 (cited within results paragraph) are already included in the net analysis presented in Reference 21.
